# Artificial Anti-HIV-1 Immunogen Comprising Epitopes of Broadly Neutralizing Antibodies 2F5, 10E8, and a Peptide Mimic of VRC01 Discontinuous Epitope

**DOI:** 10.3390/vaccines7030083

**Published:** 2019-08-06

**Authors:** Andrey P. Rudometov, Anton N. Chikaev, Nadezhda B. Rudometova, Denis V. Antonets, Alexander A. Lomzov, Olga N. Kaplina, Alexander A. Ilyichev, Larisa I. Karpenko

**Affiliations:** 1 State Research Center of Virology and Biotechnology “Vector”, Koltsovo, Novosibirsk Region 630559, Russia; 2 Institute of Molecular and Cellular Biology of the Siberian Branch of the Russian Academy of Sciences, 8/2 Lavrentiev Avenue Novosibirsk, Novosibirsk 630090, Russia; 3 Institute of Chemical Biology and Fundamental Medicine of the Siberian Branch of the Russian Academy of Sciences, 8 Lavrentiev Avenue, Novosibirsk 630090, Russia

**Keywords:** artificial protein, polyepitope B- and T-cell HIV-1 immunogen, epitopes of broadly neutralizing HIV-1 antibodies, peptide mimic of discontinuous epitope, immunogenicity

## Abstract

The construction of artificial proteins using conservative B-cell and T-cell epitopes is believed to be a promising approach for a vaccine design against diverse viral infections. This article describes the development of an artificial HIV-1 immunogen using a polyepitope immunogen design strategy. We developed a recombinant protein, referred to as nTBI, that contains epitopes recognized by broadly neutralizing HIV-1 antibodies (bNAbs) combined with Th-epitopes. This is a modified version of a previously designed artificial protein, TBI (T- and B-cell epitopes containing Immunogen), carrying four T- and five B-cell epitopes from HIV-1 Env and Gag proteins. To engineer the nTBI molecule, three B-cell epitopes of the TBI protein were replaced with the epitopes recognized by broadly neutralizing HIV-1 antibodies 10E8, 2F5, and a linear peptide mimic of VRC01 epitope. We showed that immunization of rabbits with the nTBI protein elicited antibodies that recognize HIV-1 proteins and were able to neutralize Env-pseudotyped SF162.LS HIV-1 strain (tier 1). Competition assay revealed that immunization of rabbits with nTBI induced mainly 10E8-like antibodies. Our findings support the use of nTBI protein as an immunogen with predefined favorable antigenic properties.

## 1. Introduction

Although Human Immunodeficiency Virus (HIV-1) is one of the best-characterized viruses, there is no efficient vaccine against this pathogen so far. Giving credit for notable progress in approaches to antiretroviral therapy that considerably prolongs the lifespan of HIV-infected patients, it should be noted that these are only palliative means to control the virus which cannot stop the HIV-1 pandemic [1,2]. For the most effective control of HIV-1 spread, a prophylactic vaccine should be used widely [3,4]. However, vaccine development is associated with particular well-known issues. First of all, HIV-1 genetic and consequent antigenic drift allows for evasion of the protective effects of the immune system. Therefore, traditional vaccine strategies have failed to protect against the virus [5,6,7].

Development of artificial polyepitope HIV-1 immunogens using a broad range of protective B- and T-cell epitopes from the viral antigens that can induce broadly neutralizing antibodies and responses of cytotoxic (CD8+ CTL) and helper (CD4+ Th) T-lymphocytes is one of the promising strategies for antiviral vaccine design [6,8,9,10,11,12,13].

There are a number of efforts developing artificial polyepitope T-cell immunogens [10,14,15,16,17,18,19,20,21]. Some of them have proven successful in inducing CD4+ T-cell and CD8+ T-cell responses of much greater breadth and magnitude in non-human primates compared to the vaccines containing full-length HIV protein genes [6,10]. Several polyepitope T-cell vaccine candidates have undergone phase I clinical trials [22,23,24].

The development of artificial B-cell HIV-immunogens, including those constructed using epitopes of broadly neutralizing HIV-1 antibodies (bNAbs), is the most complicated problem, since the majority of them recognize conformational epitopes and, significantly more rarely, linear epitopes. Furthermore, conformational B-cell epitopes on HIV surface glycoproteins are formed by lipids and glycans and their combinations [25,26], which further complicates the design of immunogens capable of inducing the required B-cell response. This task is believed to be solved using peptide mimics of conformational epitopes that can be obtained using combinatorial biology (the phage display technique) [27].

Concerning studies related to the development of artificial B-cell immunogens, a protein scaffold approach should be mentioned. Such scaffolds can expose one or several epitopes of broadly neutralizing antibodies to provide the most efficient exposure of the desired epitopes to the immune system [28,29,30,31,32]. Epitope scaffolds developed by rational design were able to elicit 4E10 and 2F5-like antibodies in laboratory animals [28,29]. Zhu et al. proposed computationally designed epitopes that mimic carbohydrate-occluded neutralization epitopes (CONEs) of Env through ‘epitope transplantation’, in which the target region is presented on a carrier protein scaffold. Although a tested anti-CONE serum demonstrated a modest magnitude of inhibitory activity on HIV-1 infectivity, the consistency of the effect against multiple isolates of HIV-1 Env pseudoviruses allows us to suggest that this approach could provide a broad neutralizing antibody response [33].

Another HIV vaccine strategy is based on the use of soluble stabilized Env trimer spikes for inducing broadly neutralizing antibodies. These trimeric antigens are comprised of cleavage products of gp120 and gp41 subunits forming a native-like Env conformation exposing vulnerable sites recognized by bNAbs [3,34,35,36]. However, as well as scaffolds, trimers could contain undesirable epitopes, diverting the protective humoral immune response [36,37]. To date, several approaches are used to decrease the immunogenicity of such epitopes [38,39,40,41].

Considering all of the above, it seems reasonable to create an immunogen which contains only the HIV-specific epitopes crucial for inducing a protective immune response. This approach focuses the immune response specifically on protective antigenic determinants and excludes the undesirable vaccine epitopes that could induce autoreactive antibodies or antibodies intensifying viral infectivity.

**Figure 1:**
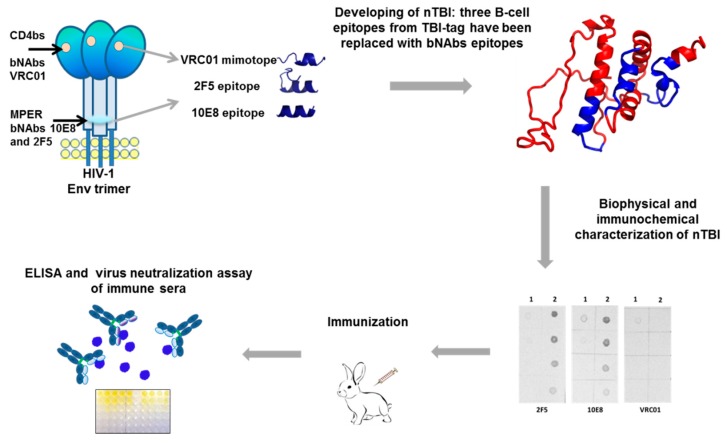
Schematic presentation of the experimental strategy for development of the nTBI protein and studying its immunogenic properties. A spatial model of the nTBI protein structure was obtained using the I-TASSER (Iterative Threading ASSEmbly Refinement) method [45].

The paper represents the results of a study on constructing and investigating immunogenic properties of an artificial nTBI molecule comprising epitopes recognized by bNAbs 2F5, 10E8 [42,43], and a linear peptide mimic of a conformational epitope recognized by VRC01 [44] (Figure 1).

## 2. Materials and Methods

### 2.1. Monoclonal Antibodies, Peptides, Bacterial Strains, Cell Lines, Plasmids, Media, and Buffers

VRC01, 10E8, and 2F5 monoclonal antibodies were kindly provided by the NIH AIDS Research and Reference Reagent Program (cat. # 12033, 12294, 1475). Murine monoclonal antibody (mAb) 29F2, *E. coli* strain BL21(DE3) pLysS (Invitrogen) and HEK 293T/17 cells (cat. # 103) as well as pTBI plasmid, encoding TBI gene (T- and B-cell containing immunogen) [46,47] were found in the collection of the State Research Center of Virology and Biotechnology Vector (SRC VB Vector, Koltsovo, Russia). TZM-bl cells (cat. # 8129) were also provided by the National Institutes of Health AIDS Research and Reference Reagent Program (USA). Synthesis of *nTBI* and *TBI_tag* genes and further cloning into pET21a expression vector (Novagen) were performed by Evrogen Lab, Ltd. (Moscow, Russia). 10E8 [NWFNITNWLWYIK], 2F5 [NEQELLELDKWASLWNK], and VRC01 [VSWPELYKWTWS] epitope peptides were synthesized by Synpeptide Co., Ltd. (Shanghai, China).

### 2.2. Expression and Purification of Recombinant Proteins nTBI and TBI_tag

The pET-nTBI and pET-TBI_tag plasmids were maintained and transformed into *E. coli* competent cells, single colonies were picked up and grown overnight in 2xYT medium with ampicillin (100 µg/ml). An overnight culture was diluted to 1:100 and grown at 37°C and 160 rpm until an OD_600_ = 0.5. Protein expression was induced with 1 mM isopropyl β-D-1-thiogalactopyranoside (IPTG). Cells were centrifuged at 6000 rpm for five minutes at 4 °C; pellets were resuspended in lysis buffer (0.05% tween 20, 50 mM monosodium phosphate, 300 mM sodium chloride, 30 mM imidazole) and sonicated on ice using Soniprep 150 Plus cell disruptor (16 times per minute at 13.2 μm amplitude). Inclusion bodies were pelleted by centrifugation, washed in Tris-HCl buffer (pH 8.3), and dissolved in 6 M urea for two hours. Insoluble fraction was removed by centrifugation, dissolved fraction was loaded onto a nickel nitrilotriacetic acid (Ni-NTA) column (Qiagen), washed with buffer containing 20 мM Tris-HCl, 0.5 M NaCl, 6 M urea and 20 мM imidazole (pH 7.9), and eluted with the same buffer with an increased concentration of imidazole up to 0.5 M. The proteins were then sequentially dialyzed for five hours at 4 °C against phosphate-buffered saline (PBS) buffer (pH 7.5) containing 6, 4, 2 and 1 M urea, respectively, followed by final dialysis in PBS. Purity and identity of refolded soluble proteins were estimated by SDS-PAGE and Western blotting.

### 2.3. SDS-PAGE, Western Blot, and New Lav Blot 1 Analysis

Protein samples were analyzed by SDS-PAGE on a 15% gel using Coomassie brilliant blue staining method. Western blot analysis was performed using SNAP i.d. system (Millipore). Proteins were transferred onto a nitrocellulose membrane (Amersham), blocked with 3% BSA for 15 minutes at room temperature, and washed three times with wash buffer (PBS with 0.1% Tween 20). After washing, the membrane was probed with mAbs VRC01, 10E8, 2F5, and 29F2 diluted to 1:10,000 in wash buffer for 10 minutes at room temperature. Antibodies were washed three times with wash buffer. Secondary antibodies (anti-human or anti-mouse) conjugated with alkaline phosphatase were used at 1:10,000, diluted in wash buffer. After 10 minutes, incubation membranes were washed five times. Proteins were visualized using nitro blue tetrazolium/5-bromo-4-chloro-3-indolyl phosphate (NBT/BCIP) substrate solution (Sigma, USA). HIV-1-specific antibodies were detected using a New Lav Blot 1 test kit (Bio-Rad, France), according to the manufacturer’s instructions.

### 2.4. Circular Dichroism 

Circular dichroism (CD) assays were carried out at room temperature (25 °C) in normal saline and in 20% trifluoroethanol using a Jasco J-600 spectropolarimeter. Spectra were recorded in the range a 195–260 nm, using a 1 mm path-length quartz cell. Each spectrum was obtained by averaging three scans with 1 nm step and 2 nm spectral bandwidth. The samples TBI_tag and nTBI have the same optical absorption at 214 nm.

The fractions of the secondary structure elements were calculated by minimizing the difference between the theoretical and experimental curves by varying of the impacts of the α-helixes, β-sheets, turns and non-structured forms. Theoretical values at every wavelength were the linear combination of the basis spectra of every type of secondary structure [48].

### 2.5. Immunization

Male Chinchilla rabbits of four weeks of age (~2 kg body weight) were purchased from Vector’s animal breeding facilities and housed in a certified animal facility managed by the Animal Center of SRC VB Vector. All experiments were made to minimize animal suffering and carried out in line with the principles of humanity described in the relevant Guidelines of the European Community and Helsinki Declaration. The protocol was approved by the Institutional Animal Care & Use Committee (IACUC) of the SRC VB Vector (# 03-02.2017).

Animals were randomly divided into two groups (three rabbits per group). The first group was immunized with the nTBI protein, the second with the TBI_tag. Immunization was carried out three times (1, 14, and 28 days). Rabbits were primed with 500 μg of corresponding proteins subcutaneously with complete Freund’s adjuvant (Sigma, USA), for the second immunization animals received 500 μg of protein subcutaneously with incomplete Freund’s adjuvant. For the third immunization rabbits were injected 500 μg of protein without adjuvant. Serum samples were collected prior to the first immunization (pre-immune) and two weeks after the third immunization.

### 2.6. Purification of Rabbit IgG

To obtain antigen-specific antibodies and to eliminate non-specific effects of other serum components pooled rabbit sera were purified using protein A chromatography (BioVision, USA). Briefly, samples (9 ml) of rabbit sera were mixed with 9 ml binding buffer (1 × TBS, 0.15 M NaCl, in 50 mM sodium borate, pH 8.0). Diluted serum samples were added to a column containing Protein A agarose equilibrated with binding buffer and passed through a column. The column was washed with 10 volumes of binding buffer. IgG was eluted with 10 ml of 0.1 M citric acid, pH 2.75, and the eluate was immediately neutralized with 1.5 M Tris-HCl, pH 8.8 (150 μl per 1 ml of eluate). For each sample, IgG fractions with the highest protein concentration were pooled and dialyzed three times at 4 °C against 1 × phosphate-buffered saline. IgG purity was assessed by the Coomassie staining of 14% SDS-PAGE gel and was found to be equal to or greater than 90%. The antibody concentrations were quantified by measuring the absorbance at 280 nm using a NanoDrop 2000 Spectrophotometer (Thermo Fisher Scientific, Wilmington, DE, USA), and then stored at – 20°C.

### 2.7. ELISA

ELISA was used to identify Ab responses of individual rabbits to nTBI and TBI_tag. MaxiSorp 96-well plates (Thermo Fisher Scientific, USA) were coated with 5 μg/ml of nTBI and TBI_tag overnight at 4 °C in PBS. Plates were blocked for two hours with 0.5% casein in PBS at 37 °C and washed three times with 0.05% Tween 20/PBS (PBS-T). Eight serial dilutions of sera samples in PBS-0.1% casein (1/200, 1/1000, 1/5000 … 1/15,625,000) were added to the wells (100 μl per well) and incubated for two hours at 37 °C with shaking. Plates were washed three times with 0.05% PBS-T. Goat anti-rabbit IgG conjugated with alkaline phosphatase (Sigma, USA) were then added to the wells (100 μl/well) at 1:5000 dilution in PBS. Plates were incubated for one hour at room temperature and washed five times with 0.05% PBS-T. To visualize immunogen-specific antibodies BCIP/NBT substrate (Sigma, USA) was added to each well and incubated overnight in the dark at 37 °C. Plates were read at 405 nm (Model 680 Microplate reader, Bio-Rad, USA). Optical density (OD) values were calculated for each group by subtracting two times the average background of pre-immune rabbit serum.

### 2.8. Production of Env-Pseudoviruses

The reference panel of recombinant plasmids containing full-length Env genes of HIV-1 subtype B [49], HIV-1 Env clone SF162.LS [50], 6535 clone 3 (Cat # 11017), QH0692 (Cat # 11018), TRO.11 (Cat # 11023), PV04 (Cat # 11022), backbone Env-deficient plasmid pSG3Δenv was obtained through the NIH AIDS Reagent Program, Division of AIDS, NIAID, NIH. Plasmids phGPMΔMD-1 encoding GP of Marburg virus were constructed at SRC VB Vector.

HIV-1 Env-pseudotyped were prepared as described in [51]. Briefly, 293T cells in a T-75 culture flask were cotransfected with 10 μg of plasmids encoding heterologous envelope variants and 10 μg of an Env-deficient pSG3 env backbone plasmid using MATra reagent (PromoKine, Germany). Pseudoviruses were collected after a 48-hour incubation, filtered through a 0.45 µm pore size filter (TPP, Switzerland) and stored at –80 °C until use. Lentiviral particles pseudotyped with Marburg envelope glycoprotein (MGP) were used as a specificity control virus in the neutralization assay. The median tissue culture infective doses (TCID_50_) of each pseudovirus was calculated by the Reed and Muench method to be used in a neutralization assay.

### 2.9. Neutralization Assay

Neutralization activities of immunogen-specific rabbit-derived antibodies were measured in the decrease of Tat-induced *Luc* reporter gene expression after a single round Env-pseudotyped HIV-1 infection of TZM-bl cells, as described previously [14,51]. Briefly, antibodies were serially diluted in a four-fold stepwise manner (initial concentration 4 mg/ml) and mixed with 50 μL of 200 TCID_50_ of pseudovirus in 96-well flat-bottom culture plates (Corning-Costar) in triplicate and incubated for 30 minutes at 37 °C. Purified IgG (initial concentration 4 mg/ml) from the pool of pre-immune sera were used as a negative control. mAbs 4E10, 2F5, and VRC01 (initial concentration 30, 25, and 10 µg/ml, respectively) were used as a positive control. The mixture was transferred to TZM-bl cells (10^4^ per well) and incubated for 48 hours at 37 °C. Following a 48-hour incubation, 150 μl of culture medium was removed from each well and replaced with a *Luc* reporter gene assay system reagent. After a two-minute incubation at room temperature to allow cell lysis, 150 μl of cell lysate were transferred to 96-well black solid plates for measurements of luminescence using a LuMate 4400 microplate luminometer (Awareness Technology Inc., Palm City, F L, USA) (expressed as relative luminescence units (RLUs) equivalent). The percentage decrease expressed in RLU (% neutralization) was determined by calculating the difference in average RLU between test wells (cells + sample + virus) and cell control wells (cells only), dividing this result by the difference in average RLU between virus control (cell + virus) and cell control wells, subtracting from 1 and multiplying by 100. To quantify neutralizing activities of rabbit antisera, the concentration of Ab required to obtain 50% neutralization (the IC_50_) was calculated. All the assays were carried out in triplicate, statistical analysis was performed using GraphPad Prism 6 software (GraphPad, USA) to calculate concentrations of antibodies (μg/ml).

### 2.10. Competition Neutralization Assay

10E8 [NWFNITNWLWYIK], 2F5 [NEQELLELDKWASLWNK], and VRC01 [VSWPELYKWTWS] peptides were synthesized by Synpeptide Co., Ltd. (Shanghai, China) with >80% purity and tested for their ability to compete with HIV-1 Env-pseudotyped virus SF162 for binding to anti-nTBI IgG, anti-TBI_tag IgG, and mAbs 10E8, 2F5, and VRC01. Competition assay was carried out as described previously [52]. Briefly, serial dilutions of Ab were tested and IC 90 values for SF162 were determined by linear regression. Next, 1:1 (v/v) mixtures of each peptide (2 mg/ml) and corresponding antibodies were incubated for 30 minutes at 37 °C, added to SF162 pseudovirus (200 TCID_50_) and coincubated for one hour at 37 °C. Further, a mix of the peptide, antibodies, and SF162 was added (1:1 v/v) to the TZM-bl indicator cell line. Luciferase activity was detected 48 hours after infection assay reagent II (Promega, USA). To control for nonspecific inhibition of the interaction between SF162 and target cells by the peptides, a neutralization assay was performed in the absence of antibodies (negative control). The inhibiting effect of peptides was detected in regards to log_10_ RLU data obtained in the neutralization of SF162 pseudovirus with respective antibodies without the addition of peptides and log_10_ RLU data obtained with the addition of peptides.

### 2.11. Statistical Analysis

A non-parametric Mann–Whitney U-test was used for multiple comparisons of the interactions between corresponding peptides and antibodies. Multiple testing correction was performed according to the Benjamini–Hochberg procedure (FDR). Statistical analysis and plotting were carried out using R, the open source statistical analysis language and environment (v. 3.3.2) [53].

## 3. Results

### 3.1. Design and Construction of Plasmids Encoding Immunogens TBI_tag and nTBI

In order to assemble the polyepitope nTBI protein, previously designed TBI (T- and B-cell immunogen) was used as a frame [46,47]. Several steps were made to improve the immunogenic properties of this modified version. Firstly, to enhance protein expression, the *TBI* gene sequence was codon optimized using the GenScript Rare Codon Analysis Tool [54]. Next, we added an *E. coli infB* gene fragment encoding N-terminal expressivity tag into the *TBI* gene’s structure [55]. The codon-optimized and expressivity tag-fused *TBI* gene was cloned into pET21a expression vector in-frame with C-terminal 6x-HIS tag, which allowed the purification of the corresponding protein by Ni-NTA chromatography. The genetic construction modified in this manner was named TBI_tag.

Next, we decided to modify the TBI_tag peptide composition by replacing three B-cell epitopes that have weak capacity to elicit virus neutralizing antibodies (according to the Los Alamos HIV Database) with the epitopes recognized by bNAbs. For this purpose, Env (255–266) and Gag (92–109) epitopes that have a predominantly alpha-helical structure were replaced with the 10E8 bNAb-specific epitopes NWFNITNWLWYIK and NEQELLELDKWASLWNK, recognized by bNAb 2F5. Both of these sequences were predicted as alpha-helical; besides, Env (255–266) and Gag (92–109) epitopes are located at the N- and C- termini of TBI, so we assumed that their replacement would not dramatically disrupt the protein backbone.

The remaining Gag (351–361) epitope had an irregular secondary structure and was surrounded by two alpha-helical regions in the context of TBI protein. Instead of this epitope, we decided to put the phage display-selected linear peptide mimic of the epitope recognized by the VRC01 antibody [52]. Similar to Gag (351–361), this peptide has an irregular structure, therefore it was assumed that incorporation of the peptide between alpha-helical motifs could give it a certain rigidity and the ability to be exposed on the protein’s surface.

**Figure 2:**
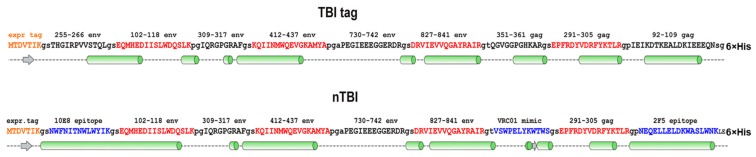
Depicting secondary structure of TBI_tag and nTBI proteins. The structures were predicted using the PSSpred tool available at [56]. bNAbs epitopes that were appended into the nTBI protein are highlighted with blue; T-h epitopes are marked with red; α-strings—light green cylinders; β-coils—grey arrows; unshaped structures—dashed lines. All proteins comprised a C-terminal sequence from histidine amino acid residues.

The proteins’ structures were predicted using the I-TASSER [45] and PSSpred [56] prediction tools, proteins models were shown in Figure 1 and Figure 2, respectively. According to modeling data, the constructed immunogens TBI_tag and nTBI have a structure of mainly α-helical regions and flexible interhelical loops.

### 3.2. Expression, Purification, and Antigenic Properties of Recombinant nTBI and TBI_tag Proteins

**Figure 3:**
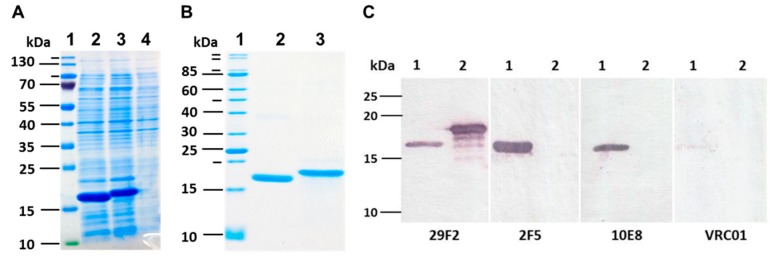
(**A**) SDS PAGE analysis of *E. coli* BL21 (DE3) pLysS cell lysate after IPTG induction. 1) molecular weight marker; 2) lysate of E. coli BL21/pET-nTBI cells; 3) lysate of E. coli BL21/pET-TBI_tag cells; 4) lysate of non-transformed E. coli BL21 cells. (**B**) SDS PAGE analysis of purified samples of nTBI and TBI_tag; 1) molecular weight marker; 2) purified nTBI protein; 3) purified TBI_tag protein. (**C**) Western blot analysis of expressed nTBI and TBI_tag. Purified proteins were separated by SDS-PAGE in 15% gel and transferred onto a nitrocellulose membrane (nTBI at lane 1 and TBI_tag at lane 2). Monoclonal antibodies 29F2, 2F5, 10E8, and VRC01 were used for immunodetection.

The designed and synthesized *TBI_tag* and *nTBI* genes were cloned in plasmid pET21a and expressed in BL21(DE3) pLysS E. coli cells. Expression of recombinant proteins encoded by the cloned genes was characterized by SDS-PAGE, followed by Coomassie blue staining (Figure 3A).

It was revealed that recombinant proteins TBI_tag and nTBI were mainly expressed as inclusion bodies; therefore, we used a purification scheme including the sonication of bacterial cells, solubilizing the inclusion bodies by urea solution, affinity chromatography using Ni-NTA resin and refolding. It was established that the modified TBI_tag can be successfully produced and purified in soluble form, while. nTBI tended to form aggregates after purification step (the solution was opalescent). The purity of isolated TBI_tag and nTBI proteins were further checked by SDS-PAGE using Coomassie blue staining (Figure 3B).

To ensure that Coomassie-stained bands correspond to the target proteins, Western blot analysis was performed using the mouse monoclonal antibody 29F2 that recognizes the peptide from HIV-1 p24 [EPFRDYVDRFYKTLR], which is a part of both nTBI and TBI-tag. It was demonstrated that both proteins specifically bind to 29F2 (Figure 3C).

To check if all the substituted epitopes retained their antigenic properties in the context of a recombinant protein, we performed Western blot analysis using 10E8, 2F5, and VRC01 mAbs as primary antibodies. It was shown that TBI_tag selectively binds to 29F2 mAb as expected, whereas nTBI reacts with all the mAbs used in screening except for VRC01 (Figure 3C).

### 3.3. Circular Dichroism

**Figure 4:**
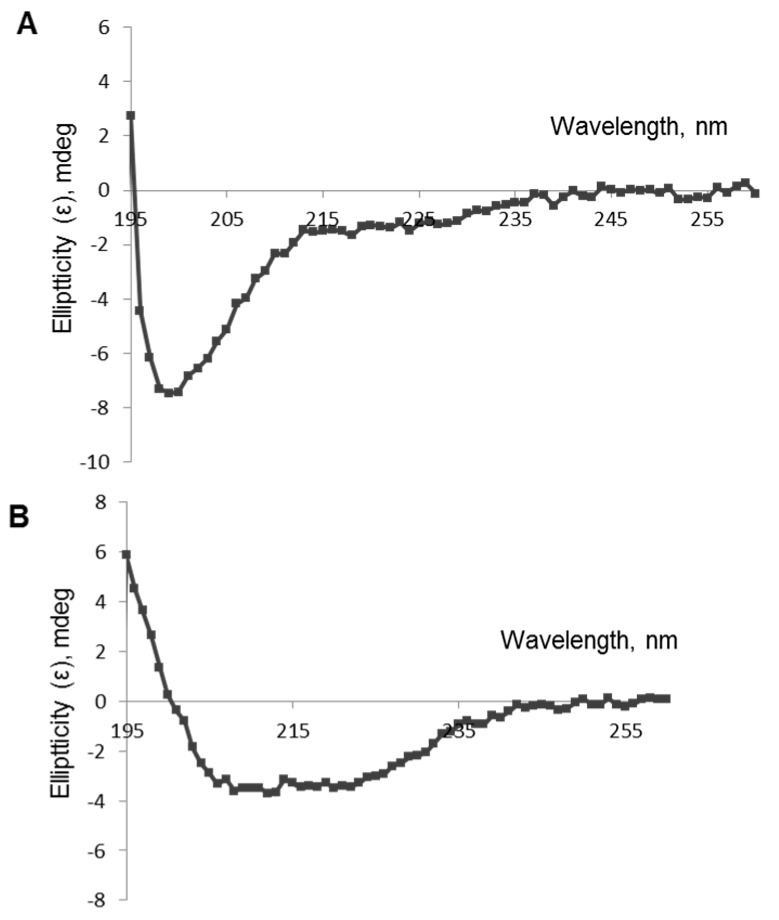
CD spectra of TBI_tag protein: (**A**) saline; (**B**) 20% trifluoroethanol in saline.

Experimental analysis of the polypeptides’ secondary structures was performed using circular dichroism spectroscopy. CD spectra of TBI_tag and nTBI were both registered in saline and in 20% trifluoroethanol in saline (Figure 4). The quantitative analysis of the secondary structure of TBI_tag shown the same fraction of α-helices and β sheets in the saline. Significant increases of α-helices and decreases of β sheets and non-structured forms were observed after the addition of 20% trifluoroethanol, which stabilizes the secondary structure of the protein (Table 1). The CD signal magnitude was close to zero for nTBI in the studied conditions.

### 3.4. Assessment of Immune Response in Rabbits

To assess the immunogenicity of recombinant proteins, ELISA was performed using sera from immunized rabbits. Pre-immune sera were used as a negative control. It was found that sera from both groups of immunized rabbits contain antigen-specific antibodies. Average antibody titers in both groups of animals immunized with TBI_tag as well as nTBI were more than 1:3,125,000 after the third immunization. Furthermore, there was no significant difference in serum level between rabbits from one group. At the same time, the signal of pre-immune sera in ELISA was no more than the background level.

**Figure 5:**
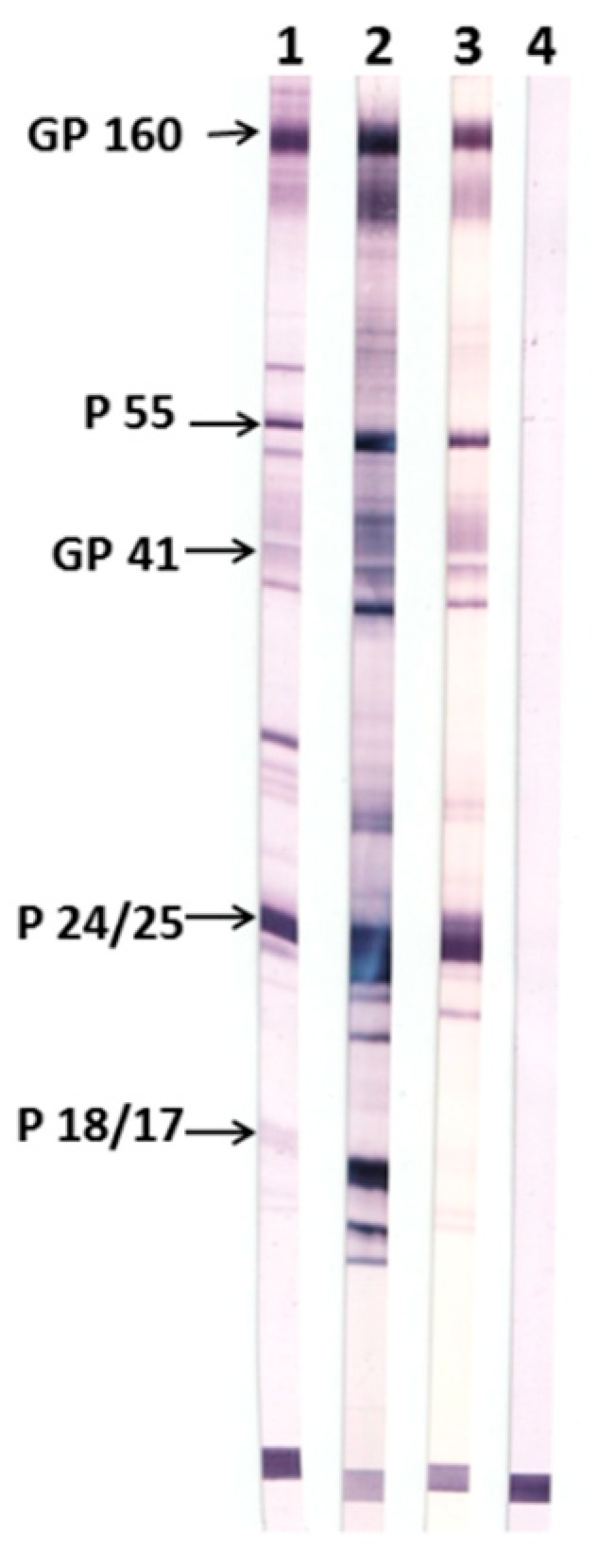
Western blot of sera from animals immunized with TBI_tag and nTBI using the New Lav Blot 1 test system. 1) Positive control from the New Lav Blot kit. 2) Pooled sera of rabbits immunized with TBI_tag. 3) Pooled sera of rabbits immunized with nTBI. 4) A pool of pre-immune sera of rabbits from both groups.

Further, sera were tested for the presence of antibodies specific to HIV-1 proteins (Figure 5). For the purpose, we used a commercially available test system, New Lav Blot 1 (Bio-Rad), including nitrocellulose stripes coated with HIV-1 proteins. Pre-immune sera were used as a negative control, while inactivated human serum containing anti-HIV-1 antibodies (from the New Lav Blot 1 kit) served as a positive control.

It was shown that antisera raised in nTBI-immunized rabbits contain antibodies against gp120, gp160, gp41, and p24 (p55), whereas TBI_tag-induced antibodies were additionally recognized p17. These results are consistent with our expectations, as nTBI doesn’t bear the p17 HIV-1 epitope which was replaced with the 2F5 epitope from the membrane-proximal external region (MPER).

### 3.5. Neutralizing Activity of Purified Specific Antibodies

IgGs purified from pool sera of rabbits immunized with TBI_tag and nTBI were tested for the capability of neutralizing HIV-1 pseudoviruses. Env-pseudotyped viruses from the global panel of reference Env clones (NIH) were packaged and used to perform a neutralization assay.

**Figure 6:**
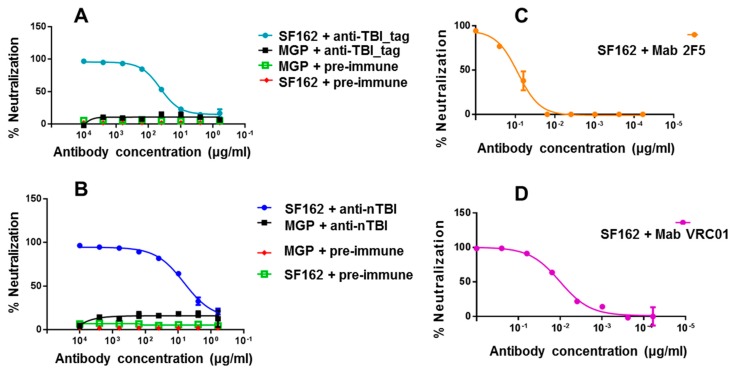
Neutralizing activity of IgGs isolated from sera of immunized animals against SF162.LS pseudovirus. The figure depicts neutralization curves for anti-TBI_tag (**A**), anti-nTBI (**B)** rabbit IgGs, as well as for human mAbs 2F5 (**C**) and VRC01 (**D**). 2F5 and VRC01 were used as positive controls. A pool of IgGs purified from pre-immune sera of rabbits (IgGs-pre-immune) was used as a negative control. Lentiviral particles pseudotyped with Marburg envelope glycoprotein (MGP) were used as a specificity control virus in the neutralization assay.

At the first step, we used easy-to-neutralize (tier 1) SF162.LS pseudovirus. It was shown that both anti-nTBI and anti-TBI_tag antibodies efficiently neutralized the tier 1 pseudotyped HIV-1 strain, while IgGs from pre-immune sera showed no neutralization activity (Figure 6). In addition, we found out that IC_50_ anti-nTBI antibodies was 0.44 μg/mL, approximately six times lower than the IC_50_ for anti-TBI_tag antibodies (2.76 μg/mL). mAbs 2F5 and VRC01 (Figure 6) were used as positive controls. Next, we performed a neutralization assay using tier 2 and tier 3 Env-pseudotyped HIV strains (6535, QH0692 и PV04). A similar trend was observed for these pseudoviruses with percent neutralization being higher for anti-nTBI IgG compared to the anti-TBI_tag IgG. However, since the mean IC_50_ values were more than 50 μg/mL, we cannot assume virus neutralizing activity relating to the used pseudoviruses.

### 3.6. Competition Neutralization Assay

Next, we decided to test whether bNAb epitopes that were included into nTBI are able to interfere with the neutralization activity of corresponding antibodies. We carried out competitive inhibition of the neutralization of the Env-pseudotyped SF162.LS HIV strain by 10E8, VRC01 and 2F5 mAbs, and purified IgG from immune sera using synthetic peptides 10E8 [NWFNITNWLWYIK], 2F5 [NEQELLELDKWASLWNK], and VRC01 [VSWPELYKWTWS].

Preliminary experiments revealed that these peptides are non-toxic for TZM-bl cells and induce no non-specific inhibition of the interaction between pseudovirus and target cells.

**Figure 7:**
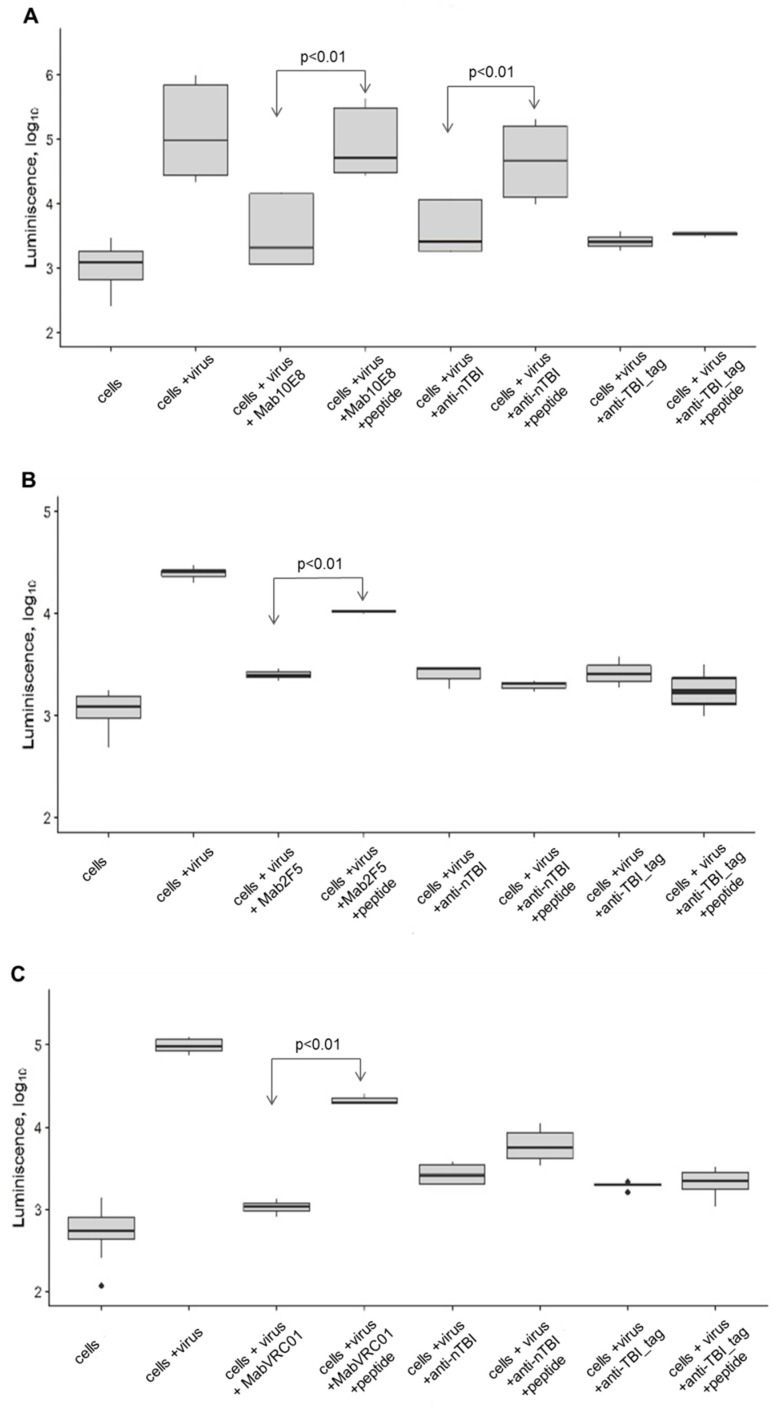
Inhibition of virus-neutralizing activity of antibodies by synthesized peptides: (**A**) 10E8 [NWFNITNWLWYIK], (**B**) 2F5 [NEQELLELDKWASLWNK], (**C**) VRC01 [VSWPELYKWTWS]. The following combinations were tested in the assay: cell + virus; cell + virus + monoclonal mAb 10E8 or 2F5, or VRC01; cell + virus + monoclonal mAb 10E8 or 2F5, or VRC01 + corresponding synthetic peptide; cell + virus + anti-nTBI IgG; cell + virus + anti-nTBI IgG + synthetic peptide; cell + virus + anti-TBItag IgG; cell + virus + anti-TBItag IgG + synthetic peptide. Relative luminescence intensity is indicated on the Y-axis (log10 RLU). Statistical differences between groups cell + virus + mAb 10E8, or 2F5, or VRC01 and cell + virus + mAb 10E8, or 2F5, or VRC01 + synthetic peptide is *p* < 0.01; statistical differences between groups cell + virus + anti-nTBI IgG and cell + virus + anti-nTBI IgG + synthetic peptide is *p* < 0.01.

10E8 peptide showed the inhibition of neutralizing activity of anti-nTBI antibodies against SF162.LS (*p* < 0.01). Additionally, inhibition of neutralization of TBI_tag-specific antibodies by this peptide was not observed (Figure 7A). Predictably, 10E8 peptide epitope blocked neutralization of SF162.LS by 10E8 (*p* < 0.01) (Figure 7A).

A peptide mimic of the VRC01 epitope inhibited neutralization of SF162 by VRC01 (*p* < 0.01), but weakly blocked neutralization of SF162 by anti-nTBI antibodies (*p* <0.20), and not inhibit TBI_tag-specific antibodies (Figure 7C). As expected, the 2F5 epitope interfered with 2F5 mAb and decreased its neutralizing activity against SF162, whereas we observed no inhibition of neutralizing activity of both anti-nTBI and anti-TBI_tag antibodies (Figure 7B).

## 4. Discussion

Eroshkin et al. have previously designed an artificial polyepitope TBI protein (T- and B-cell epitopes containing immunogen) composed of conservative epitopes from Env and Gag HIV-1 and based on a well-known protein structural motif, i.e., a four-helix bundle [47]. TBI included four Th-cell epitopes (amphipathic α-helix) and five B-cell epitopes (regions with flexible hydrophilic loops) [46,47]. The rationale for the TBI design was that combining T- and B-cell epitopes in one construct would stimulate both proper B-cell and T-cell responses and the necessary interplay between B- and T-cells. The TBI protein had a CD spectra similar to α-helical proteins and showed crystal-yielding capacity, which was demonstrated for the first time in an artificial protein with a predicted tertiary structure [57].

It was shown that TBI induced both cellular and humoral responses to HIV-1 in immunized mice and rhesus macaques, and TBI-induced antibodies showed virus-neutralizing activity to HIV-1 [58]. Lately, TBI was included into the composition of the CombiHIVvac candidate vaccine that had undergone phase I clinical trials [24,58].

Based on its ability to crystallize, we assumed that the TBI protein structure was similar to that of the natural protein. We decided to design a modified protein based on TBI with an enhanced ability to induce HIV-neutralizing antibodies.

First, in order to increase the yield of recombinant protein, we changed the expression vector to pET21a, which led to an increase of gene expression level and allowed the use of the 6xHis/Ni-NTA system for protein purification. Additionally, we added an *E. coli infB* gene fragment encoding an N-terminal expressivity tag to its structure [55]. This protein was referred to as TBI-tag and used for further modifications. At the next step, we replaced three B-cell epitopes from TBI-tag with epitopes recognized by bNAbs. We substituted the Env (255–266), Gag (99–109), and Gag (351–361) epitopes (Figure 2). The first two epitopes were replaced with peptides from the MPER region, i.e., (NEQELLELDKWASLWNK) and (NWFNITNWLWYIK), recognized by bNAbs 2F5 and 10E8, respectively. Instead of Gag (351–361), we added a phage display-selected linear peptide mimic of the epitope recognized by the VRC01 antibody, into the protein structure [52]. According to the PSSpred tool modeling data, the epitopes for 10E8 and 2F5 have retained their peculiar α-helical structure, which was shown to be required for their antigenic and immunogenic properties (Figure 2).

T-helper epitopes and spacer sequences between epitopes remained unchanged, since they form amphipathic α-helices which seem to stabilize protein structure (Figure 2). Significance of the helical amphipathicity of epitopes for the recognition of T-cells is evident, since 70% of T-helper epitopes have an α-helical conformation. Previously, it was shown that TBI elicited HIV-specific T-cell response in mice, which supported the idea that TBI processing as well as presentation of T cell epitopes were adequate [46]. Since antigen-presenting cells present T-cell epitopes as linear peptides after antigen processing, we suppose that the functional activity of T-helper epitopes in the context of nTBI should have remained at about the same level as in TBI.

Modeling the secondary structure enabled us to assume that accomplished modifications didn’t significantly affect the recombinant protein organization, since the predicted secondary structures of TBI_tag and nTBI were found to be similar (Figure 1 and Figure 2).

To evaluate the secondary structure of the TBI_tag and nTBI proteins, we used a circular dichroism spectroscopic method. CD spectra analysis of the proteins in a 20% trifluoroethanol solution revealed that TBI_tag contains 54% α-helices and 4% β-sheets, which is consistent with theoretical data predicted with PSSpred; 52% and 2%, respectively. The corresponding predicted values for nTBI were the following: 59% of α-helices and 3% of β-sheets. However, we failed to determine its secondary structure using CD. The probable reason is that the inclusion of the peptide mimic of the VRC01 conformational epitope into an internal area of the molecule TBI_tag resulted in a destabilizing of the protein structure and changing of its physico-chemical properties, since it became prone to aggregation. Western blot analysis revealed that the mAb VRC01 poorly interacted with the peptide mimic as part of nTBI as compared to the same peptide as part of protein p3 of bacteriophage M13 [52]. Two other peptides, 2F5 and 10E8, included in the nTBI molecule are effectively recognized by their corresponding MPER bNAbs (2F5 and 10E8) (Figure 3A).

Antibody titers of rabbits’ antisera derived after three-fold immunization with both TBI-tag and nTBI were more than 1:3,125,000, which indicates the high immunogenicity of both proteins. New Lav Blot 1 analysis showed that induced antibodies in rabbits by immunization with TBI_tag and nTBI were capable of binding to HIV-1 proteins (Figure 5). Consequently, both immunogens were able to elicit HIV-specific antibodies.

The analysis of virus neutralizing activity of sera from animals immunized with both TBI_tag and nTBI revealed that both proteins elicit antibodies capable of neutralizing tier 1 SF162.LS. Additionally, anti-nTBI IgG neutralizing activity proved to be higher than that of the antibodies induced with TBI_tag. The IC50 of anti-nTBI antibodies was six times lower than the IC50 for anti-TBI_tag antibodies (Figure 6). To test whether the neutralizing activity of anti-nTBI IgGs from rabbits’ immune sera is mediated by the antibodies which were elicited against substituted epitopes, we performed a peptide competition neutralization assay.

The 10E8 peptide was the most immunogenic, since its inhibition capacity of anti-nTBI IgG significantly differed from control (Figure 7A). Thus, enhancement of virus neutralizing activity of antibodies induced by nTBI seems to be connected to the inclusion of peptide 10E8 in the compound of the TBI protein.

VRC01 mimotope VSWPELYKWTWS contributes poorly to the induction of neutralizing antibodies. When immunized with the nTBI protein, the neutralizing activity of the antibodies induced against this mimotope decreased after the addition of the VSWPELYKWTWS peptide; although the difference between the control group was not significant (Figure 7C). Probably, this may be explained by a shielding of the peptide mimic by flanking amino acid sequences in the nTBI compound. Thereby, placing the peptide mimetic of the VRC01 epitope into the internal region of nTBI could alter the immunogenicity of this peptide. Importantly, the peptide mimic of the VRC01 epitope efficiently inhibited the binding of bNAbs VRC01 to the SF162 pseudovirus, which is consistent with the literature [52].

Concerning 2F5 peptide, we failed to demonstrate inhibition of SF162 neutralization by IgGs from rabbits immunized with TBI_tag and nTBI. The epitope recognized by the bNAb 2F5 was designed at the protein C-terminus, and was followed by an additional six histidine residues. Inclusion of this hexahistidine tag helped streamline protein purification, yet it could also affect the structure and accessibility of the adjacent epitope. This could be one of the possible reasons why the 2F5 epitope retained antigenicity in the context of the nTBI, but failed to induce 2F5-like Abs (as shown by the competition analysis using the synthetic 2F5 peptide). It seems that 2F5 epitope heavily relies on its natural environment to expose its immunogenic features [29]. The obtained findings will be considered in the further enhancement of TBI immunogen. It is possible that removing the histidines from the C-terminus of nTBI will result in efficient presentation of the 2F5 peptide.

Another possible explanation of low neutralizing activity of the immune sera could be the animal model that we used for the experiment. Although rabbits can be used for characterization of HIV-1 immunogenicity, they are not a valid model for the analyses of novel immunogens driving an anti-HIV response. One reason is that they are naturally resistant to HIV infection, and more importantly, sequences of their immunoglobulin genes are too diverged from those of primates. In our future experiments testing the elicitation of neutralizing antibodies, common marmosets will be used as the model animals.

## 5. Conclusions

Approach to the development of artificial proteins constructed using conservative T- and B-cell epitopes and their mimics is believed to be promising for the development of vaccines against variable viruses, including HIV. In such constructs, all of the component helper T cell epitopes may be expected to provide T cell help for antibody production against all of the component B cell epitopes, thus overcoming the limitation of genetic restriction [46]. In theory, this approach makes it possible to evade the antigenic variability of the virus, focus the immune responses on protective determinants, and exclude from the vaccine compound undesired determinants capable of inducing autoantibodies or antibodies increasing virus infectivity.

In this study, we used a polyepitope-based HIV immunogen design strategy to develop an artificial nTBI protein exposing epitopes recognized by bNAbs 2F5, 10E8, and a phage display-selected peptide mimic of the VRC01 discontinuous epitope. Antigenic properties of the incorporated peptides were saved in the compound of nTBI, except for the peptide mimic VRC01. nTBI became less soluble as compared to the initial TBI. This is probably because substitution of the initial TBI B-cell epitopes by new ones negatively affected its conformation, despite the prediction results. However, compared to the initial TBI, nTBI demonstrated a better capacity to induce virus neutralizing antibodies in comparison with the initial TBI, at least for the SF162 pseudovirus the neutralizing activity of nTBI-induced IgGs outperformed that of the TBI_tag-induced IgGs (the IC50 of anti-nTBI antibodies was six times lower than the IC50 of anti-TBI_tag antibodies (Figure 6)). Competition assay revealed that immunization of rabbits with nTBI induced mainly 10E8-like antibodies, whereas no 2F5 epitope and VRC01 mimotope-induced antibodies were detected. We considered several strategies for boosting the antigenicity and immunogenicity of nTBI. The peptide mimetic targeted by VRC01 can be placed at the C- or N- terminus of TBI, which may translate into the induction of neutralizing antibodies. Our earlier results [52] indicate that the peptide mimetic selected using phage display was able to induce neutralizing antibodies, either in the context of the M13 phage or as individual synthetic peptide. Furthermore, following affinity selection, we obtained many more VRC01 mimetics, and these may be considered viable alternatives for future modifications of TBI.

As for the 2F5 epitope, testing whether the C-terminal hexahistidine tag affects its immunogenicity remains an attractive experiment to do. Also, one may think of increasing the spacers between the epitopes, as this may translate into greater accessibility of the epitopes to B-cell receptors. Establishing a broader range of TBI derivatives may therefore be highly productive, as this may help identify the variants showing greater activity. Finally, the lead immunogen should be thoroughly tested in various prime-boost immunization schemes. The ultimate goal of this approach is to obtain an anti-HIV vaccine component that would provide the desired neutralizing immune response. It is possible that such an immunogen will comprise a cocktail of several lead TBI versions.

## Figures and Tables

**Table 1:** Circular dichroism spectra of the TBI_tag protein.

Secondary Structures	Saline	20% Trifluoroethanol
α-helices	13%	54%
β-sheets	14%	4%
Turns_I	2%	4%
Unordered structures	70%	37%
